# Decision-making process for introduction of maternal vaccines in Kenya, 2017–2018

**DOI:** 10.1186/s13012-021-01101-7

**Published:** 2021-04-12

**Authors:** Nancy A. Otieno, Fauzia A. Malik, Stacy W. Nganga, Winnie N. Wairimu, Dominic O. Ouma, Godfrey M. Bigogo, Sandra S. Chaves, Jennifer R. Verani, Marc-Alain Widdowson, Andrew D. Wilson, Irina Bergenfeld, Ines Gonzalez-Casanova, Saad B. Omer

**Affiliations:** 1 Division of Global Health Protection, Centre for Global Health Research, Kenya Medical Research Institute, PO Box 1578-40100, Kisumu, Kenya; 2 Hubert Department of Global Health, Rollins School of Public Health, Emory University, 1518 Clifton Rd NE, Atlanta, GA 30322 USA; 3 Centre for Global Health Research, Kenya Medical Research Institute, PO Box 1578-40100, Kisumu, Kenya; 4 National Center for Immunization and Respiratory Diseases, Centers for Disease Control and Prevention, PO Box 606-00621, Nairobi, Kenya; 5 Division of Global Health Protection, Center for Global Health, Centers for Disease Control and Prevention, PO Box 606-00621, Nairobi, Kenya; 6 Department of Medicine, Division of Pediatrics, Emory University School of Medicine, 1518 Clifton Rd NE, Atlanta, GA 30322 USA

**Keywords:** Maternal vaccines, Decision-making, Policy formulation, Policy implementation

## Abstract

**Background:**

Maternal immunization is a key strategy for reducing morbidity and mortality associated with infectious diseases in mothers and their newborns. Recent developments in the science and safety of maternal vaccinations have made possible development of new maternal vaccines ready for introduction in low- and middle-income countries. Decisions at the policy level remain the entry point for maternal immunization programs. We describe the policy and decision-making process in Kenya for the introduction of new vaccines, with particular emphasis on maternal vaccines, and identify opportunities to improve vaccine policy formulation and implementation process.

**Methods:**

We conducted 29 formal interviews with government officials and policy makers, including high-level officials at the Kenya National Immunization Technical Advisory Group, and Ministry of Health officials at national and county levels. All interviews were recorded and transcribed. We analyzed the qualitative data using NVivo 11.0 software.

**Results:**

All key informants understood the vaccine policy formulation and implementation processes, although national officials appeared more informed compared to county officials. County officials reported feeling left out of policy development. The recent health system decentralization had both positive and negative impacts on the policy process; however, the negative impacts outweighed the positive impacts. Other factors outside vaccine policy environment such as rumours, sociocultural practices, and anti-vaccine campaigns influenced the policy development and implementation process.

**Conclusions:**

Public policy development process is complex and multifaceted by its nature. As Kenya prepares for introduction of other maternal vaccines, it is important that the identified policy gaps and challenges are addressed.

**Supplementary Information:**

The online version contains supplementary material available at 10.1186/s13012-021-01101-7.

Contributions to the literature

- This research systematically describes the policy and decision-making process for introduction of new vaccines, with particular emphasis on maternal vaccines.
- According to our findings, public policy process is complex and multifaceted in its nature and needs to be transparent and involves all key stakeholders.
- The research identifies policy gaps, challenges with policy processes, and opportunities for improving vaccine policy formulation and implementation process.
- As the maternal vaccine pipeline expands, the opportunities identified for improving vaccine policy process will help in informing coordinated, more inclusive, and better understood policy-making process for smoother implementation of maternal immunization programs in low- and middle-income countries.

## Introduction

Maternal immunization is a key strategy for reducing infectious disease morbidity and mortality in mothers and their newborns [1]. Most countries recommend immunizations during or prior to pregnancy as part of a cost-effective public health strategy [2, 3]. Most high-income countries recommend immunization of pregnant women for tetanus, influenza, and pertussis [4], while low- and middle-income countries (LMICs) recommend immunization of pregnant women for tetanus and, in some cases, influenza [5, 6]. Despite recommendations for maternal vaccines and accumulating evidence on their safety and effectiveness in preventing diseases, there is still a lag in uptake. Although coverage of tetanus vaccination among pregnant women has been optimal in most countries, coverage of influenza and tetanus diphtheria and acellular pertussis (Tdap) remain variable [2]. In fact, influenza and Tdap are yet to be incorporated as part of maternal vaccination policies in many LMICs; this is due to limited local evidence to inform decisions on vaccine introduction [7]. Moreover, new vaccines (e.g., against Respiratory Syncytial Virus and Group B *Streptococcus*) are being developed for use in pregnant women. Decisions at the policy level remain the entry point for new maternal immunization programs. Therefore, it is critical to address barriers to acceptance prior to introduction as the maternal vaccine pipeline expands.

In Kenya, tetanus toxoid (TT) is the only maternal vaccine approved by the Kenya Expanded Program on Immunization (KEPI); however, coverage is low with only 50% of women receiving two or more TT doses in their last pregnancy according to the last Kenyan Demographic and Health Survey [8]. Recent efforts by the government to improve TT uptake were challenged by religious groups (the Catholic Church) that questioned whether the vaccine contained human chorionic gonadotropin, a birth control hormone [9]. The concerns were escalated through mass media and took government policy makers’ involvement to effectively address the concerns in a collaborative way. Hence, Kenya faces challenges of balancing efforts to address barriers towards the only approved maternal vaccine, while putting in place strategies for introduction of other maternal vaccines. The objective of this study was to describe the policy and decision-making process in Kenya for the introduction of new vaccine recommendations, with emphasis on the maternal vaccination program, and to identify opportunities to improve the vaccine policy formulation and implementation process.

## Methods

### Study design

We conducted qualitative semi-structured interviews on perspectives on maternal vaccination, with healthcare providers and pregnant women from four counties in Kenya [10], and with policy makers and implementers at national and county levels. In addition to this, we carried out a cross-sectional survey of knowledge, attitudes, and beliefs regarding vaccination among pregnant women [11] and healthcare workers [12], as part of a larger multi-study project that aimed to create an evidence base for determinants of maternal immunization acceptance to inform policy makers and implementers on future maternal vaccination strategies in Kenya. In this paper, we present data from qualitative interviews with national and county officials.

### Study setting

We implemented the study in five sites located in Marsabit County Referral Hospital, Coast Provincial General Hospital (Mombasa County), Tabitha Clinic Kibera and Mbagathi District Hospital (Nairobi County), and Siaya County Referral Hospital and Lwak Mission Hospital (Siaya County) representing the four broad geographical settings in Kenya including the North Eastern, Coastal, Central, and Western regions of the country. These sites were included based on geographic diversity, poor immunization coverage, and high burden of maternal and infant mortality [13].

The Kenya Medical Research Institute (KEMRI) and the Centers for Disease Control and Prevention–Kenya (CDC) have a long history conducting population-based surveillance for infectious diseases, sentinel surveillance for influenza, and health and demographic surveillance system [14–16] in these areas. Through these platforms, KEMRI and CDC have developed close working relationships with national and county Ministry of Health (MoH) management teams.

### Sample

Key informants at the MoH national level included Kenya National Immunization Technical Advisory Group (KENITAG) core members, MoH heads of divisions for Family Health and the National Immunization Program (NIP), and managers of different units at the NIP involved with advocacy, communication and social mobilization, vaccine logistics, and monitoring and evaluation. At the county level, key informants included Chief Officers of Health and managers of divisions covering maternal and child health, policy, health promotion, and monitoring and evaluation activities (Table 1).

**Table 1:** Policy makers and implementers interviewed

Officer interviewed	Office level	Number interviewed
Deputy director public health	County	1
Deputy director preventive and promotive services	County	1
Deputy director reproductive health	County	1
Head of National Immunization Program, MoH	National	1
Officer in charge of expanded program on immunization logistics	County	5
Nursing officer, in charge of reproductive health and maternal and child health	County	4
Head, Division of Family Health, MoH	National	1
Health promotion officer	County	4
Deputy chair, KENITAG	National	1
Core member, KENITAG	National	3
Officer in charge of monitoring and evaluation	County	2
Officer in charge of advocacy, communication, and social mobilization	National	1
Nursing officer	County	1
Officer in charge of community strategy	County	1
Officer in charge of vaccines locally & internationally	National	1
Public health coordinator	County	1
Total	County	29

### Participant identification

Division heads at the MoH and the KENITAG were approached to help with identification of national officials at the MoH’s divisions for Family Health and the NIP and core members of KENITAG who had experience in immunization issues in Kenya.

We approached chief officers and county directors for health to help with identification of county officials working within departments for maternal and child health, policy, health promotion, vaccine logistics, and monitoring and evaluation. Upon identification of the county officials, each official was approached, and appointments were made for interviews at a date, time, and place most convenient to their schedules.

### Data collection at national and county levels

The interview guide was developed using grounded theory methodology (with questions added to the interview guide as insights were gained from initial interviews done) by the lead study anthropologist with input from study investigators, scientific advisory committee, and the study team [10]. The guide was pretested on two county level officials in charge of immunization logistics and one community health nurse, the guide was revised to bring into clear focus questions around demand creation strategies for maternal vaccines, barriers, and drivers for maternal vaccine program implementation in Kenya and recommendations for improving the vaccine policy-making process.

Study staff received protocol-specific training, additional training on qualitative data collection procedures and tools, and on interviewing techniques. Interviews were carried out from April to September 2017. Using a semi-structured interview guide, we administered open-ended questions that guided discussions on topics covering factors associated with vaccine recommendations, professional experiences with evidence-based policy-making, and historical shifts in maternal health and/or vaccine policy. The interviews were conducted in English, tape recorded, and transcribed verbatim at the end of the interview day. Staff also took notes during discussion for additional information that may be required during analysis. Each interview lasted about 1h, participation was voluntary, and no compensation was provided to the participants. The interviews/discussions were concluded once all the key informants targeted at national level were interviewed and upon attaining data saturation for county-level informants.

### Data management and analysis

We used both framework (code frame) and thematic approaches in this analysis. The research team developed a codebook for the qualitative data from the interview guide questions. We used both structural and content coding techniques to develop our codebook and to describe our resulting thematic observations, while making comparisons for consistency among four members of the coding team to ensure high inter-coder reliability (at kappa > 0.8 on 10% of transcripts). We analyzed the data using NVivo 11.0 software for qualitative data analysis, driving the data analysis while utilizing deductive methods for thematic and pattern identification. We categorized codes to fit into a coding frame where related codes were merged into sub-themes and matching sub-themes finally merged into the main themes.

## Results

We conducted a total of 29 interviews (8 from national and 21 county levels). The themes that emerged from the analysis entail policy development and implementation process, effects of decentralization (referred to as ‘devolution’) on healthcare policies, resilience of policy to external influences, and recommendations for improving vaccine policy-making process. The themes and sub-themes are illustrated in Table 2.

**Table 2:** Summary of themes and sub-themes from data analysis

Themes	Sub-themes
Decision-making process for policy development and implementation	- Problem identification - Considerations for vaccine introduction
	- Monopolized engagement of national level entities in vaccine policy recommendation
	- Complexity of decision-making process
	- Policy implementation process (systematic implementation)
	- Policy evaluation
Effects of devolution on health care policies	- Policy adaptation to local needs
	- Intra-governmental communication
	- Delineation of responsibilities
	- Resource provision within government
Resilience of policy to external influences	- Rumours
	- Anti-vaccine campaigners
	- Sociocultural influences
Recommendations for improving vaccine policy-making process	- Stakeholder engagement at all levels - Improve intra-governmental communication

We highlight some quotations from key informants in the results. Additional quotations are summarized in Tables 3, 4, 5, and 6.

### Policy development and implementation: decision-making process

#### Problem identification

Kenya has not introduced any new maternal vaccination policy since the adoption of the 5 TT vaccination schedule in 2002. However, in the last few years, several new vaccines for children have been licensed (e.g., pneumococcal conjugate vaccine (2011) measles second dose (2013), rotavirus (2014), injectable polio vaccines (2016), etc.), all of which are implemented by KEPI. The majority of the key informants shared that in general, vaccine policy formulation is driven primarily by the presence of a problem to be addressed.

*“First, there must be a problem for us to have a policy to work towards that problem. Maybe we have done a situation analysis and found an area that has a gap. We usually call in stakeholders to deliberate with us on the issue during policy formulation.”* (R05, national level)

Although vaccine policy formulation was thought to be primarily driven by internal recognition of a problem, most vaccine policy formulation in Kenya has resulted from global recommendations by international organizations such as the World Health Organization (WHO) or availability of international funding for vaccine introduction. Local evidence was sought only in response to external recommendations as the country tried to fit into the global agenda.

*“… I think by and large for the government, it is almost as if we do not have that response where the local research is informing and driving the policy agenda. It is sort of like it is reversed where we are fitting in the global agenda which is related to high level discussion where you may be want to eradicate or eliminate a disease, or introduce this vaccine because it has been introduced elsewhere and there is funding that can sponsor the introduction and all that. We do not have that where our own research is driving the policy but maybe with time we will get there.”* (R03, national level)

#### Considerations for vaccine introduction

Our discussions with stakeholders with experience in health policy formulation revealed important considerations that have guided policy formulation for introduction of new infant vaccines in the country (Table 3). The main issues included disease burden, vaccine effectiveness, safety, cost-effectiveness of vaccine introduction, and sustainability of the vaccination programme once it has been rolled out.

*“The first thing is it must be safe. …what it protects from should be more than what it could cause us because vaccines also have their negative impacts…*

*It should also be able to do what we are saying it can do.*

*…It should be cheap for us to be able to buy it because if it is too expensive it is of no use…*

*Let me say it should be accessible meaning if I want to buy it as an individual, the little I have should be able to buy it.*

*…Safety, effectiveness, accessibility and other factors along that line*.” (R06, national level)

#### Monopolized engagement of national level entities in vaccine policy recommendation

Most of the policy makers at the national level cited the involvement of the KENITAG in the technical review of information upon situational analysis and problem identification. KENITAG is composed of senior members of the relevant medical specialities (adult medicine, paediatrics, immunology), experts from the universities (epidemiology, public health, microbiology, pathology and law), and relevant personnel from MoH (*ex-officio* members), or non-government organizations (liaison members).

*“The next level after the above considerations is the technical phase. This is the level at which we look at the considerations with an inquisitive and critical mind. … This is where Kenya National Advisory Group on immunization (KENITAG) comes in. This is a technical group to which we forward all the gathered information and let them discuss logically. We do this because they have technical knowledge of each aspect. KENITAG comes up with recommendations as advisories to each aspect of the policy dimension.”* (R04, national level)

The role of KENITAG in policy recommendation was most comprehensively explained by one of the core members involved in advising and capacity building at the MoH for vaccine-related matters (Table 3). Created in 2014, this relatively new group has matured with time; though it is challenged by various requests from MoH and with no budgetary allocation for operational activities such as regular meetings to facilitate policy discussions. Guided by an internal procedures manual, KENITAG has a standard and stepwise approach for developing independent evidence-based recommendations. The group has made recommendations for four different vaccines, involving Measles, Mumps, and Rubella; Human Papilloma Virus; influenza (infants aged 6–23 months); and review of safety data for TT.

**Table 3:** Policy development and implementation; decision-making process

Sub-theme	Quotes
*Problem identification*	*First you need, first we do an analysis, situation analysis, to identify whether there’s a need or if there’s a gap. Once we identify if there’s a gap for this policy, specific policy, we write to our permanent secretary... We make a request to start the process of developing a policy towards the either maternal or child health... Once we get that approval, we constitute a steering technical committee that understands very well on that area that will work on that mmmmmm policy document. And there will be steering meetings to inform and in those, there will be public participation, there’s partners’ participation. So for maternal policy I think, applies just like any other, any other policy, in the ministry or the division. (R05, national level)*
*Considerations for vaccine introduction*	*We have epidemiological data which guides the ministry on the rationale behind introducing vaccines. We also have general directions globally from the World Health Organization of what is required. We also use authority bodies like the Pharmacy and Poisons Board where we get licensed vaccines into the country. We do all these in consultations with research institutes such as KEMRI polio lab and KEMRI measles lab. Collating this data helps us to tell the worth of a vaccine and its cost. (R01, national level)* *We must also consider our capacity to deliver the vaccines. We have to determine the side effects of the vaccine as well. You understand that vaccines are just like any other drug with side effects. We have to establish that the benefits outdo side effects. We also take into consideration the population which is at risk. What is the magnitude of the burden of diseases in question? (R02, national level)* *Well, of course involves both the division of disease surveillance and the division of vaccines at national level. I have not been engaged in the policy development process but the best of my knowledge at county level is we would look at disease prevalence, we would look at some of the conditions that are affecting our mothers, we would look at availability of vaccines that are effective globally, uh WHO …WHO approved and then look at possibly what is the cost of that disease …uh in terms of life, …so the Disability Adjusted Life Years (DALYs) …look at the cost of the vaccine and then do a cost effectiveness analysis. (R05, county level)* *The need for a vaccine is driven by certain factors about diseases. For example, the prevalence of the diseases, ability to control the disease, is the vaccine available, is the vaccine desirable, safe and all other basic sciences about the vaccine. However, bottom line is that after doing baseline issues such as the need for a vaccine, the cost is always a factor. (R07, county level)* *The first thing is it must be safe. Its pros must outdo its cons; what it protects from should be more than what it could cause us because vaccines also have their negative impacts…* *It should also be able to do what we are saying it can do. Safety alone does not guarantee that it is effective…* *Safety, effectiveness, accessibility and other factors along that line. (R06, national level)*
*Monopolized engagement of national level entities in vaccine policy recommendation*	*When the government has an intention of rolling out a vaccine.* *… they seek justification first to decide for or against rolling out a vaccine. We (KENITAG) therefore, adduce the evidence and submit it to the ministry by collating information and discussing it with the aim of unearthing the pros and cons. These will lead us into making recommendations based on scientific facts, our own studies and experiences. We collate information from all these sources and pass it to our client, ministry of health. They do not have to take our advice. (R07, national level)* *KENITAG is comprised of experts in different fields. These include pediatricians, researchers and professors who look at the diseases and they are able to delve deeper. They find out more issues about the disease to create understanding that helps shape the direction of a vaccine policy. They also look at the justifications for introducing a vaccine or not. (R04, national level)* *The final decision is at the discretion of the government since it considers several angles. On our part, we are guided by the scientific dimension of the vaccines. (R07, national level)* *Since we are mostly at the county level, we pick the national policy and domesticate it at our level; we rarely come up with our own policy… We then call our stakeholders, pick the policy issues relevant to us from the national policy, and put in our own policy document. (R20, county level)*
*Complexity of decision-making process*	*…before it (vaccine policy) reaches endorsement, there is a lot of technical deliberations and fact finding about the vaccine. As a unit, we (Unit of Vaccines and Immunizations) are mandated to take charge of that. I can therefore say that there is no policy which can be made without our involvement. We are the only program (National Immunization Program) with the authority to handle immunization issues in the country. (R08, national level)* *It is the cabinet secretary of health who makes the final decision. He has his technical officers at the Division of Vaccines and Immunization (DVI) who advice the cabinet secretary (CS) who then pass that on the decisions. Even as they deliberate on that, there are financial implications which the CS must consider when tabling vaccination recommendation at the cabinet. There must be a round table where you explain the money to be allocated. (R07, county level)*
*Policy implementation process (systematic implementation)*	*Once the policy has been drafted, tried and passed, then it is the ministry to cascade it down. Remember we now have a devolved system with forty-seven governments. The bigger role of the central government is policy-making then cascade it down to the devolved units to make it in to practice. (R03, national level)* *The ministry makes policy documents to the people who will implement the policies. (R16, county level)* *What I have seen is there is a launch, normally there would be a launch, an introductory launch. At county level we normally would get sensitized, invited to the launch get a sensitization package on the new policy, get introduced to it and then the national would actually roll it out to us then it would be our responsibility at the county to ensure that we have implemented that policy. So what would happen is we would have meetings with our sub-county teams, sub-county would have meetings with facilities introduce that then would see how to now roll it out to the community with the sensitization through our community health structures and through our health promotion officers and ensure that the information goes round but the implementation really falls on us at the county, how we do procurement, what we administer then would be at the county level. (R20, county level)*
*Policy evaluation*	*We also support the counties with policy guidelines on immunization in soft and hard copies for their reference during practice. We also organize several trainings, so that we update new issues, discuss observed challenges and possible solutions. (R02, national level)* *…as a country, and as a county, we have adopted the performance approach. So each person has their set targets, so if you don’t meet, your supervisor will ask you why haven’t you met your targets, you will explain. (R08, county level)*

*“One of the terms of reference is that we advise the ministry of health on all matters relating to vaccines and immunizations and this involves introduction of new vaccines or even modification of existing vaccine schedules… Those are basically our roles in NITAG, mainly related to making evidence-based recommendations for vaccines and immunizations.*

*… we have a document called the Internal Procedures Manual that was developed by all the KENITAG members.*

*…The procedures manual is our day-to-day document that guides the operation of KENITAG and inside there it clearly spells out the process for making vaccine policies.”* (R07, national level)

Very few policy makers were aware of the process for policy changes for vaccine introduction, which involved approval for policy change by the National Immunization Interagency Coordinating Committee (NIICC). This body comprises MoH technical partners and expert representatives from international partners, and ensures that KEPI gets financial support both locally and internationally to run its operations. The approval is done after stakeholder engagement at different levels when KENITAG recommendations are discussed.

*“These are then brought back to Division of Vaccines and Immunization (DVI) where we engage widely and at all levels of policy-making. We are responsible for engaging all relevant bodies and organizations to have a thorough discussion on the policy. We engage different levels of leadership and dimension; political level, technical level and potential obstacles among others.*

*…After engaging all relevant leadership and organizations, we then put in a request to have the vaccine introduced and to go through. Just before the request is made, we have the National Immunization Interagency Coordinating Committee which provides oversight and gives the approval for the policy change for the introduction of the vaccine.”* (R08, national level)

As much as policy makers at the national level shared that the policy formulation was an all-inclusive process involving stakeholders from different fields and at different levels, the majority of key informants at the county level felt left out of the policy development process. They reported engagement only during policy implementation process.

*“Like we know the policies are made at the national level … we also have our policy makers...They should also be able to participate and also, some technical persons from county level.* (R01, county level)

*…so most of the policies we disseminate at the county have been developed at the national office.”* (R02, county level)

#### Complexity of the decision-making process

**Fig. 1:**
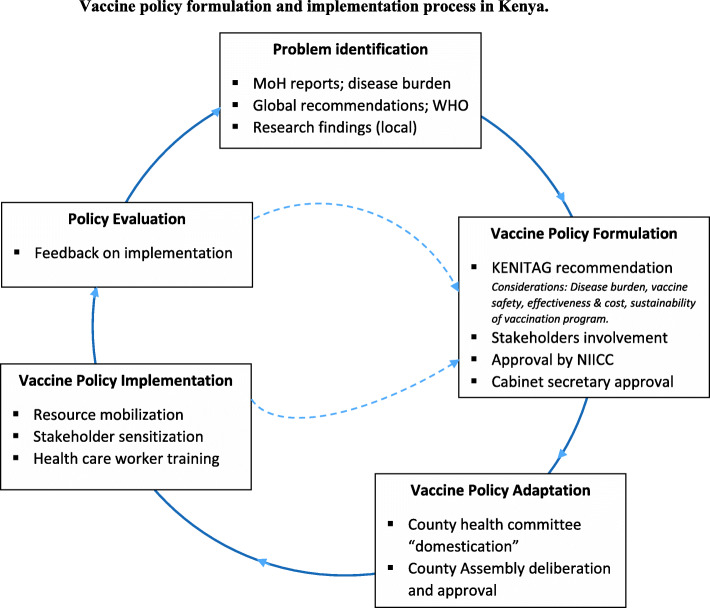
**Vaccine policy formulation and implementation process in Kenya.** Note: *KENITAG*, Kenya National Immunization Technical Advisory Group; *MoH*, Ministry of Health; *NIICC*, National Immunization Interagency Coordinating Committee. Domestication refers to the process of deliberating on the national policy document by county committees and coming up with a county policy document (adaptation) in line with the national policy. National policy processes involve problem identification, policy formulation, and policy evaluation. County policy processes involve vaccine policy adaptation. Shared policy process between national and county: vaccine policy implementation. Dotted lines are feedback loops illustrating information flow for issues arising during policy implementation and evaluation processes, communicated back to national level for consideration or action

One perception that was evident across all informants was that the country had no specific maternal vaccine policy and introduction of new maternal vaccines would borrow from existing national policy guideline on immunizations in general. Policy-making was viewed as a lengthy process involving information gathering from various sources and intensive consultations with various stakeholders working together (Fig. 1).

“*The policy-making process is lengthy…*

*Considering vaccines, if we wanted to come up with any vaccine to be included in the schedule then we must ensure that the whole broad area of vaccination information is available. That means that all vaccines now and in the future must be taken care of since vaccines are bound to increase.”* (R06, county level)

#### Policy implementation process (Systematic implementation)

Mobilization of resources for policy implementation was a prerequisite once the policy was approved, upon which relevant stakeholders were engaged to strategize on implementation. Policy directives were disseminated from the national to the county levels and cascaded down to the facility level, and eventually to the target population (Table 3).

*“In terms of implementation, it is the mandate of Unit of Vaccines and Immunization (UVI) or Division of Vaccines and Immunization (DVI) (now referred to as the National Immunization Program) to ensure operationalization of the policy. This is done through capacity building to cascade it to the lower levels using the governance in place in the health system. We are the central level and the next level to cascade the program would be the county. At the county, we have the County Health Management Team. We then roll it to sub-county where we have Sub-county Health Management Team who then roll it out to facilities. That is when the common mwananchi (Swahili term for citizen) as we call them in Kenya gets the vaccines.”* (R02, national level)

Very few informants at the county level understood the information delivery process for vaccine policy from the national level. The few who understood the process explained that the communication came down through the council of governors to the county directors for health.

*“I; who receives instructions from the national level?*

*R; The national level has to write to the council of governors, the council of governors will write to the governor, who will action his county executive committee member for health who will write to the chief officer and director to implement, and at the end of it then the county logistician under my department (preventive and promotive services) will actually be responsible and of course under the directive of the director to implement… we will look at what are the instructions and make sure, of course still in consultation with the national office, it meets the needs of the county.”* (R16, county level)

Policies were not just adopted as received; the national policy document was reviewed by relevant county health/policy committees, to pick policy issues relevant to county needs. The counties then came up with their own policy document, but in line with the national directives, a process termed as adaptation or domestication of national policy to county policy. The document was approved before final implementation at health facility level.

*“We pick the national policy and domesticate it at our level; we rarely come up with our own policy. We then call our stakeholders, pick the policy issues relevant to us from the national policy, and put in our own policy document. We have both political and general stakeholders, who must buy in to our policy... we take the policy to the county assembly for approval, after which we give it to the executive at the county level to approve. The policy document is launched once it has been approved at the county level.”* (R20, county level)

#### Policy evaluation

Evaluation forms a critical component of intervention implementation. While very few informants from the county were aware of efforts by the national government to evaluate vaccination programs that had been rolled out in the recent past or enforce vaccine recommendation, officers at the NIP reported giving technical support to county governments during vaccine policy implementation. Moreover, accountability for implementing vaccine policy recommendations by health providers was mainly mentioned by county health managers.

*We conduct support supervision and technical visits to the county, sub-county and the health facilities. We also go to the community so that we are able to get information from the real beneficiaries to get their view about the service which we are providing. From there we are able to cover information on new policy if there are. If there is anything we need to correct that is the place to do it.”* (R02, national level)

The vaccine policy formulation and implementation process in Kenya is summarized in Fig. 1, illustrating the sequential process. Once a problem was identified, a policy was formulated to address the gap. The policy discussions involved different stakeholders at the national level. The policies were then passed down to the county level for review and adaptation to suit local needs, before implementation. In the event that significant issues emerged with the policy during implementation, feedback was communicated back to the national level for review. Similarly, feedback from the policy evaluation process was communicated to the national level. If any changes were made to the policy following feedback from the implementation and evaluation processes, the changes were passed back to the county and a similar sequential process was followed for review, adaptation, and implementation, as a new policy.

### Effects of devolution on health care policies

#### Policy adaptation to local needs

While policy formulation involved officers at the national level, when policy directives were rolled out to counties, the county governments had the opportunity to review and deliberate on the national policies through their county health committees as provided for by county government regulations.

*“The county works within committees such as health and policy committees. We use the health committee when we want to talk to the assembly. There is a committee that brings all the committees of the county assembly together, where members of the health committee are also members there. They deliberate and approve it first, and then we take it to the governor’s office where the last approval happens then we launch the document.* (R12, county level)

*…We find very many gaps in the national policy because they do not realize how unique Nairobi is. We fill those gaps and domesticate it for ourselves.”* (R21, county level)

#### Intra-governmental communication

Introduced in 2013, the devolved system of government came with challenges as there was not clear transition to the new county system of some functions that were originally within the mandate of the national government (Table 4). This resulted in communication gaps with many county officers reporting poor communication between the national and county governments and therefore a lapse in information sharing from national to county level. These lapses in communication trickled down to frontline workers who were not adequately informed on updated immunization guidelines.

*“So sometimes forums are lacking to sensitize all the leadership so that we are on common page, you know.* (R02, county level)

*But importantly you also need to look at the health care workers who’ll be the people administering the serum. They also need to be sensitized and be updated on that.”* (R18, county level)

#### Delineation of responsibilities

Absence of clear county health department/division structures and clarity of roles in the devolved system were reported. The counties were not aware of the division of responsibilities between national and county governments, and this slowed down policy implementation process for most health interventions and services.

*“So that now with the devolution, I mean we are a little bit on breakdown between who is-who is to do what between the county government and the national government… personally I would say it has taken time for Nairobi as a county, the health sector and generally the county, to take up its rightful role in allocating the required resources for vaccine.”* (R07, county level)

#### Resource provision within government

Gaps still existed between what the national government provided before devolution and what the county government could now provide (Table 4). Inadequacy of critical resources for vaccine policy implementation (such as information education and communication materials and supplies needed for vaccine delivery and administration) hindered the implementation processes.

**Table 4:** Effects of devolution on health care policies

Sub-theme	Quotes
*Policy adaptation to local needs*	*The county works within committees such as health and policy committees. We use the health committee when we want to talk to the assembly. There is a committee that brings all the committees of the county assembly together, where members of the health committee are also members there. They deliberate and approve it first, and then we take it to the governor’s office where the last approval happens then we launch the document. (R12, county level)*
*Intra-governmental communication*	*…regular updates on the KEPI schedule because we’ve been introducing several vaccines in the last three years but there’s been no trainings going on the schedule, the complete schedule. So we are training on specific antigen. … before, it was very clear if they were 9 they were 9 and everyone knew they were 9 now people don’t know if they are 10 or 20 but they know there is something, yeah they brought something ‘ya’ (for) diarrhoea, there is something ‘ya’ (for) pneumonia but nobody is talking about the package… ok we have introduced fine, but what is the package? …information is scanty. (R04, county level)*
*Delineation of responsibilities*	*“With the vaccine policy of I think 2013, the national government is supposed to procure the vaccines and the county government is supposed to implement and make sure people get the vaccines or the vaccines get to the end users. There have been many challenges with the devolved system because there was a year we did not have budget allocation for procuring vaccines, all the money went to the counties, and when it reached there, it could not go back [to national procurement]. I think there is a gentleman’s agreement where the money to procure the vaccine remains at the national level, but I am not sure the counties even have the budget to administer the vaccines and take them to the community.” (R07, county level)* *…initially the funding for everything would come from Nairobi, but now we are finding that the funding has to come from the county. Now at the county level, to access now the funding, now it has become a problem. (R17, county level)*
*Resource provision within government*	*Proper planning and resource mobilization is also critical, we have had some challenges in the past. Yes, it’s important especially for resources… like when we were doing the switch for the OPV (oral polio vaccine) vaccines we didn’t have enough resources, when we were introducing rotavirus the resources came way later so it posed a challenge for us. (R03, county level)* *Lack of IEC (information, education and communication) materials, I don’t know the last time we had IEC materials on vaccines so it is very difficult to educate communities without that. (R06, county level)* *…of course we’ll always say resource limitation, as much as now we have a budget on EPI it is still very limited, it is less than 1% of the total county budget on health. (R11, county level)* *When it comes to vaccine distribution, we are talking about transport, in terms of net cost, we are talking about storage facilities for the cold chain of the vaccines, which this year I’m aware, Nairobi County has budgeted for procurement of cold chain equipment like fridges and other equipment, bearing in mind that vaccine program was run at the national level, and the county at the county level, it has not been fully taken over as a county function in terms of allocation of resources. (R07, county level)*

*“…sometimes there is stock out of the vaccine and that’s basically because of the transportation chain and also the procurement chain. Sometimes we may miss some of the logistics. …like either syringes, ice packs.”* (R13, county level)

### Resilience of policy to external influences

Several factors outside the policy system impacted both the formulation and implementation systems and called for stakeholders to devise coping mechanisms.

#### Rumours

Both policy makers and implementers cited rumours by certain interest groups as the major factor that interfered with vaccine rollout in the country, for new vaccines and even for existing vaccines during catch-up campaigns (where vaccination would target people who missed some scheduled vaccine doses or did not complete a vaccine series) (Table 5).

**Table 5:** Resilience of policy to external influences

Sub-theme	Quotes
*Rumours*	*The Roman Catholic Church has given us issues concerning vaccines. There are certain events that occurred two to three years ago and that has given us a problem with vaccination. The aspect of getting the Jesuits on board is very important. They are the scientific wing for the Catholics. (R05, national level)* *Yes, but then those health professionals have their beliefs as well. There is this group called Opus Dei. They are learned too. However, they do not want to hear about vaccination. Community attitudes to certain vaccines like TT and the idea that it is contraception and that is also a religious organization propagating this I think due to miscommunication. (R07, national level)*
*Anti-vaccine campaigners*	*Yes, we are there (anti-vaccine campaign) just that the degree is different. It is not just in vaccination but also in medicine. There are issues around traditional herbs and there are people who want us to go back to that. It all starts as a small religious learned group of a sophisticated learned class. They argue that we have lived like that before and it can be done again. They can be quite influential. (R05, national level)* *The key point is addressing their concerns in timely and proper manner because a lot of it is misinformation and misdirected journalists who write materials with the commercial aim and not necessarily from professional point of view.* *Part of the effort is therefore to have scientific writers who can be critical in journalism. (R10, county level)*
*Sociocultural influences*	*The other thing is belief that some of these diseases have solutions within the cultural setup and remedies are not found on conventional medicine. Therefore, people believe the diseases do not exist or are just results of bad omen. It is like when you talk about neonatal death for example. When a child dies at three days old, you hardly notice a funeral activity for the child… yet it can be neonatal tetanus which can be prevented through vaccination with tetanus vaccine. To them, the death can be related to other cultural issues such as ‘in my lineage first born children have to die and it is normal’. …What is important is understanding the vaccine and culture around people. (R19, county level)* *In-deed culture has a role to play. Everything has its base in culture because your mother is more powerful even when you go to school or not. Even if you have gone to school, there is no much difference between what you do and what she believes. If you are talking about culture in relation to pregnant mothers, we can consider Traditional Birth Attendants which we are trying to play with now. (R21, county level)* *We cannot rule out culture since it is part of us. Those doing anthropology and sociology study patterns of resort and how people behave; even health seeking behavior which are entrenched in culture. Therefore, you cannot ignore that. However, education can solve certain cultural beliefs. For example, someone may believe that smearing cow dung on the cut umbilical cord can fasten healing process while education tells you that is the most effective way of contracting tetanus. Some of them can be mitigated. (R14, county level)*

*“Back here in Kenya we had controversies with tetanus vaccine. The church and the state had a standoff in that the former claimed the vaccine caused sterilization in children. We had misinformation about the cervical cancer vaccine given to girls and not boys…”* (R08, national level)

Controversies surrounding tetanus vaccine were the most cited interferences to maternal vaccine campaigns. The situation was further complicated when the sources of misinformation were reported to be well-educated people and professionals who were respected in society.

*“There are rumors spread all over that the vaccines are laced with family planning. …If the doctors themselves are outright Catholics and oppose some of the vaccines. If there are staunch Catholics who sit on a panel to discuss vaccines and they say no to a vaccine they will be believed.” (R05, national level)“Anti-vaccine” campaigners*

#### “Anti-vaccine” campaigners

Anti-vaccine campaigns emerged as a significant factor that was growing in Kenya (Table 5). The increased access to social media in the country in the recent past further facilitated the spread of anti-vaccine perspectives and therefore their influence.

*Vaccine hesitancy is not just a Kenyan case but world over. People who are against vaccines are so audible yet it is a small group in the country…With IT (information technology) and communication technology make it easy for information to flow. People who are pro vaccine talk less more loudly as opposed to those who are against it. The latter amplify their information and cause problems in the country*. (R08, national level)

#### Sociocultural influences

Government officials have learned to prepare in advance for external influences on policy processes (Table 5). Anticipating sources, pre-empting misinformation, engaging policy processes groups that had potential to propagate misinformation and addressing concerns were reported as some of the strategies that have worked well to counter vaccine-related rumours and misinformation.

*“So I think you must address those type of concerns [vaccine side effects] before you're asked. … preempt those things, answer them, and then give the information to the public before you just say, take this germ...*

*Therefore, it is timeliness, accurate information and less use of press. There must be proper use and handling information professionally so that consumers are not confused.”* (R10, county level)

### Recommendations for improving vaccine policy-making process

#### Stakeholder engagement at all levels

For better understanding of the need for vaccine introduction, key informants at the county level highlighted the need for their involvement in decision-making at the inception stage (Table 6). This would help ensure an effective implementation process when the policy was rolled out. Other important stakeholders identified by informants included lobby groups that would influence public opinion, and therefore, their buy-in was considered critical for acceptance of vaccine at the community level.

**Table 6:** Recommendations for improving vaccine policy-making process

Sub-theme	Quotes
*Stakeholder engagement at all levels*	*I have one recommendation that in policy-making process all stakeholders must be engaged. By stakeholders I mean right from those benefiting from the service and the service providers. Therefore, to me it is team work and you need everybody with an interest on board so that when decision is taken, it is implemented together. (R09, county level)* *The beneficiaries of a policy are important in policy making process. If you ask me, I would be more direct with health providers as well. They are the entry point and should form part of policy making particularly when the vaccine is not surrounded by controversy. These are facilitators who can also be pushback factors when there are controversies. (R03, national level)* *It is important to get buy in from communities and opinion leaders to avoid resistance over misinformation, that is the greatest lesson I think that has really come out, because you can have all good intentions but if the opinion leaders do not understand then there is miscommunication then you get very low uptake. (R10, county level)* *So actually involving the, the entry point to any introduction of any antigen also helps a lot. Because if you avoid some people somewhere down the ground, they can be a roadblock and then you find that you don’t succeed. (R15, county level)*
*Improve intra-governmental communication*	*Engage the counties more, we are in a new system of devolution so I think moving forward we need to have more engagement. We have seen some attempts …but increasing the engagement. (R17, county level)* *I think one of the lessons has been being inclusive. We must be inclusive in passing of information. (R01, county level)*

*“Well first, from the inception of that vaccine, all the stakeholders must be involved. Now that we are devolved, we are told that the national level prepares the policies. I’d wish, even if they have to pick one person per county to go and take part so that we feel involved… not just as a way of communicating your decision, but communicating to them the essence of introducing this and showing the need of having these vaccines… So that as this policy comes down, we are able to, take it up.”* (R09, county level)

#### Improve intra-governmental communication

Nearly all informants expressed the need for a refined communication process between national and county governments as policy formulation was a consultative process and needed feedback on the implementation process, too. It was also considered important that a clear communication process was maintained among county leadership and between county leadership and frontline workers.

*“But for the TT that time, only two persons, that was the director and nursing officer, were called to a meeting in Nairobi [on TT catch-up campaigns]... and it will run concurrently with the polio. So the larger team were fully involved in the polio, but the TT was left for the two to run. So the TT was not well pronounced… in terms of preparation, … advocacy, … social mobilization, it was never well planned, never well executed.”* (R11, county level)

## Discussion

This study had four main findings. First, while all key informants understood the vaccine policy formulation and implementation processes, national officials appeared more informed compared with county officials, with only officers at the NIP mentioning the role of NIICC in these processes. Second, county officials felt excluded from policy development processes and were only involved when vaccine policies were ready for roll-out. Third, policy makers identified positive and negative impacts of devolution on policy formulation and implementation process. Lastly, there were factors outside policy development and implementation that influenced vaccine-related policy including public opinion, interest groups, and lobbying, among others.

The majority of the informants were well versed with important considerations that guide decisions for vaccine introduction in Kenya. These topics were in line with the key areas recommended by WHO for deliberation before deciding to introduce a vaccine to a national immunization program [17]. These include disease concerns (public health priority, magnitude of disease burden, availability of other prevention, and control strategies), vaccine concerns (safety, performance, cost, and cost-effectiveness and affordability), and capacity of immunization program/health system (successful vaccine introduction and sustainability of delivery). Even though the policy makers and implementers identified the vaccine policy process, most vaccine introductions have been based on international recommendations by WHO [18–20]. Of interest, in policy formulation, was the independent technical advisory role by KENITAG that was relatively new yet critical in vaccine introduction as it promoted evidence-informed immunization policy recommendations. The process of evidence-based policy-making is a new concept and will change as Kenya experiences introduction of new vaccines.

Policy formulation is a lengthy process that entails bringing together different stakeholders, consultations, making evidence-based decisions guided by studies and different information sources [21–23]. While the MoH officials at the national level reported stakeholder inclusion at all levels of vaccine policy development, the county officials felt excluded from the policy formulation processes, yet they were expected to implement the guidelines. It is useful, however, to engage those involved in the process of developing and implementing new vaccine policies at all levels [24]. Authors of a study in Uganda evaluating the prioritization of new vaccines in low-income countries emphasized the importance of a transparent and all-inclusive participatory process involving stakeholders at the national and sub-national levels, including the public, for improved decision-making process and successful introduction of new vaccines [25]. Other LMICs have demonstrated successful inclusive policy process, for example, India where policies have been successfully formulated at local government level for introduction of new vaccines with support from the national government [26]. In Bangladesh, researchers observed an all-inclusive policy process where the national government was the main actor in vaccine policy-making decisions, but with involvement of different stakeholders and partners [27]. Of note, as much as Kenya county government officials felt excluded from policy development process, it is important that stakeholders understand their roles in policy development and implementation process. Policy development is largely a function of the national government, while implementation is a county function. Some county presence in policy development is, however, critical for representation of county perspectives and needs and for eventual smooth translation of policy upon dissemination from the national level. Within this effort, it is essential to consider representation of stakeholders at county and lower levels as they similarly have decision-making roles in adaptation of policies disseminated from the national level, and for smooth implementation of policy at the grassroots level.

In 2013, the governance system in Kenya changed from a centralized to a devolved government of 47 counties where health functions are managed by county governments. The process of vaccine policy dissemination from the national to county level was not clear to the majority of informants at the county level. Despite this, upon receipt of the policy directives, county-level officials embraced and adapted the policies to ensure they met their local needs. Similarly, the county officials did not understand the role of the national government in evaluation of vaccine policy to assess whether policy directives were implemented or identify challenges that arose with implementation. The NIP has a unit that monitors and evaluates vaccine and immunization activities.

The decentralization of government functions had effects on policy development, implementation, and evaluation processes. The devolved system of government significantly increased the decision-making space at the county level, where counties deliberated on national policy documents and developed county policies in line with national directives. On the downside, some of the health sector functions were transferred to the county faster than most of the health sector workers had anticipated [28], and most counties had not put in place structures to undertake the functions. This should have improved with time as counties set up their structures; however, county officials still reported unclear delineation of responsibilities and resource provision between national and county governments 5 years after implementation of the devolved system. Moreover, county officials were mostly unaware of government policy proceedings and were often notified at the time of implementation without being consulted about the resources available or the needs observed at the county level. Given that devolution involved transfer of power of managing health resources from the national to county level, it is not surprising that there was disconnect in information sharing within the government.

Public policies are influenced by a number of factors including public opinion, interest groups, lobbying, and new scientific discoveries, among others. These factors tend to push and pull policy in different directions thereby slowing the development and implementation of new policies [22]. Rumours have negatively affected vaccine policy implementation worldwide for several years. The rumours are usually festered by a combination of circumstances and factors, for example, people encountering bad experiences with government health interventions, political tensions linked to presidential elections, and confirmation of rumours by influential or highly placed religious and political leaders who give the rumours credence. Some of these have included sterilization fears linked with tetanus vaccination in early 1990s that disrupted tetanus vaccine programmes in many countries [29]. In Nigeria, five states boycotted polio vaccine due to fear that it was unsafe, which slowed down the country’s progress towards polio elimination [30]. Similarly, rumours involving tetanus and polio campaigns have affected the uptake of the vaccines in East Africa [31]. In our analysis, there was a recurring theme of rumours interfering with vaccine policy implementation. The majority of the informants having had first-hand experience in handling misinformation that tetanus vaccine, used by the Kenyan government during campaigns in selected parts of the country in 2014, was laced with a family planning hormone. The fact that the sources of the information were members of a well-educated group (medical practitioners) within the Catholic church had a greater influence on the lay populace.

This study had a few limitations. Key informants at the county level were from only four (albeit diverse) of Kenya’s 47 counties. We ensured, however, that the proportion of key informants were equally distributed across the four counties. Although the information shared by the officials from the four counties included was quite consistent, it may not adequately represent the perspectives of other counties. Finally, most of the data collection was in reference to a hypothetical vaccine introduction among pregnant women.

This is the first study to explore the policy decision-making process in the introduction of new vaccines in Kenya to help understand how vaccines are adopted, rolled-out, and monitored, and examine challenges with decision-making process and vaccine delivery. Understanding motivations behind policy-level decisions affecting the introduction of new maternal vaccines is important, particularly in a low-resource country such as Kenya, with multiple competing public health priorities [32]. Results from this study can be useful to improve the process of vaccine policy development and vaccine introduction efforts in Kenya and similar settings.

## Conclusion

The maternal vaccine pipeline continues to expand, as maternal immunization remains an important strategy for reducing morbidity and mortality from vaccine preventable diseases in mothers and newborns. Kenya does not have a procedure specifically for developing vaccination policy for maternal vaccination: although a framework that has been used for introduction of other vaccines exists. Policy formulation processes mainly involved officials at the national level despite the devolution of health system in Kenya. There were some efforts to include counties in these decisions; the efforts evidently remained suboptimal, and the system had information gaps with the county officers lacking clear understanding of policy processes, while they were expected to implement the policies. Public policy process is complex and multifaceted by its nature and needs to be transparent and involve all players. As Kenya strategizes for introduction of maternal vaccines, it is important to identify policy gaps and challenges that can be addressed for a coordinated, more inclusive, and better understood policy-making process, and smoother implementation of maternal immunization programs.

## Supplementary Information

**Additional file 1.** COREQ Checklist.

## Data Availability

Data supporting the findings of this study are available from the corresponding author upon reasonable request.

## Abbreviations

CDC: Centers for Disease Control and Prevention
CS: Cabinet secretary
DVI: Division of Vaccines and Immunization
IEC: Information, education and communication
IT: Information technology
KEMRI: Kenya Medical Research Institute
KENITAG: Kenya National Immunization Technical Advisory Group
KEPI: Kenya Expanded Program on Immunization
MoH: Ministry of Health
NIICC: National Immunization Interagency Coordinating Committee
NIP: National Immunization Program
OPV: Oral polio vaccine
Tdap: Tetanus diphtheria and acellular pertussis
TT: Tetanus toxoid
WHO: World Health Organization
